# Knowledge, health beliefs and attitudes towards dementia and dementia risk reduction among descendants of people with dementia: a qualitative study using focus group discussions

**DOI:** 10.1186/s12889-021-11415-2

**Published:** 2021-07-07

**Authors:** J. Vrijsen, E. L. M. Maeckelberghe, R. Broekstra, J. J. de Vries, A. Abu-Hanna, P. P. De Deyn, R. C. Oude Voshaar, F. E. Reesink, E. Buskens, S. E. de Rooij, N. Smidt

**Affiliations:** 1 Department of Epidemiology, University of Groningen, University Medical Centre Groningen, Hanzeplein 1, FA40, PO Box 30 001, 9700 RB Groningen, the Netherlands; 2 Wenckebach Institute for Training and Education, University of Groningen, University Medical Centre Groningen, Groningen, the Netherlands; 3 Department of Genetics, University of Groningen, University Medical Centre Groningen, Groningen, the Netherlands; 4 Department of Neurology and Alzheimer Centre Groningen, University Medical Centre Groningen, Groningen, the Netherlands; 5 Department of Medical Informatics, University of Amsterdam, Amsterdam UMC, Amsterdam, the Netherlands; 6 Department of Psychiatry, University of Groningen, University Medical Centre Groningen, Groningen, the Netherlands; 7 Department of Internal Medicine & Geriatrics, University of Groningen, University Medical Centre Groningen, Groningen, the Netherlands

**Keywords:** Focus groups, Qualitative research, Primary prevention, Dementia, Attitude, Health beliefs, Awareness, Knowledge, Risk reduction behaviour, Dementia risk reduction, Life style

## Abstract

**Background:**

Individuals with a parental family history of dementia have an increased risk of developing dementia because they share their genes as well as their psychosocial behaviour. Due to this increased risk and their experience with dementia, they may be particularly eager to receive information regarding dementia risk reduction (DRR). This study evaluated the knowledge, beliefs and attitudes towards dementia and DRR among descendants of people with dementia.

**Method:**

Using a semi-structured topic guide, three focus group discussions were conducted consisting of 12 female (80%) and 3 male (20%) descendants of people with dementia with a mean (± SD) age of 48.8 (± 12) years. Focus group discussions were audio recorded and transcribed. Each transcript was analysed thoroughly, and where appropriate, a code was generated and assigned by two researchers independently. Then, similar codes were grouped together and categorized into themes.

**Results:**

The items in the topic guide could only be addressed after participants had been given the opportunity to share their experiences of having a parent with dementia. Participants were unaware or uncertain about the possibility of reducing the risk of developing dementia and therefore hesitant to assess their dementia risk without treatment options in sight. Moreover, participants indicated that their general practitioner only gave some information on heritability, not on DRR. Although participants identified a large number of modifiable risk factors as a group during the group discussions, they were eager to receive more information on dementia and DRR. In the end, participants adopted a more positive attitude towards a DRR programme and provided suggestions for the development of future DRR programmes.

**Conclusions:**

Although the research aim was to evaluate the knowledge, beliefs and attitudes towards dementia and DRR, sharing experiences of having a parent with dementia seemed a prerequisite for considering participants’ own risk of developing dementia and participating in a DRR programme. Knowledge of dementia and DRR was limited. Due to unawareness of the possibility of reducing dementia risk, participants were hesitant about assessing their dementia risk. Group discussions positively changed the perception of dementia risk assessment and participants’ willingness to participate in a DRR programme.

**Supplementary Information:**

The online version contains supplementary material available at 10.1186/s12889-021-11415-2.

## Background

Dementia is an age-related multifactorial disorder, and a growing body of evidence reveals that the risk of developing dementia later in life is determined by the co-occurrence of non-modifiable risk factors (e.g., apolipoprotein e4, family history) and modifiable risk factors across one’s lifespan [1–3]. Over the last decade, evidence of modifiable risk factors for dementia has been mounting [1–3]. The Lancet Commission on Dementia Prevention, Intervention and Care demonstrated that 40% of dementia cases are attributable to twelve modifiable risk factors (i.e., less education, hearing loss, midlife hypertension, midlife obesity, smoking, depression, physical inactivity, diabetes, low social contact, excessive alcohol consumption, traumatic brain injury, and air pollution) [3–5].

Nevertheless, it is a challenge to enable individuals to change their health behaviour to tackle modifiable risk factors. Several behaviour change theories explain the determinants of health behaviour change, of which the health belief model (HBM) is believed to be the best suited model for dementia risk reduction [6–8]. Nonetheless, there is a consensus on four major constructs to measure the motivation to change one’s lifestyle and health behaviours, including 1) knowledge of the disease and its risk factors; 2) perceived severity of the disease; 3) perceived susceptibility of the disease; and 4) motivation, including perceived benefits or barriers to performing risk-reducing behaviour [6, 8]. Given that descendants of people with dementia have experience with dementia, they might be particularly eager to receive information and obtain more knowledge regarding dementia risk reduction. Moreover, descendants of people with dementia might be receptive to adopting a healthier lifestyle to reduce their dementia risk.

A recently updated review by Cations et al. (2018) summarized the evidence of previous surveys on the knowledge of dementia and dementia risk reduction [9, 10]. The included studies were conducted in the general population in Europe, the US, Eastern Asia, Israel, and Australia and found that knowledge about the opportunity for dementia risk reduction is poor but may be improving over time [9, 10]. However, these studies’ data were often collected through surveys, whereas qualitative data collection through focus groups might be more useful to obtain insight into the beliefs and attitudes towards dementia and dementia risk reduction. The open structure of focus group discussions provides the ability to identify unanticipated themes [11]. Kim et al. (2015) conducted a focus group study to investigate the knowledge, beliefs and attitudes towards dementia and dementia risk reduction in the general population aged 50 years and older [12]. They found that both fear of developing dementia and the need to improve dementia knowledge are important motivators for adopting and maintaining a healthier lifestyle for dementia risk reduction [12]. To our knowledge, none of the previous studies were aimed at a selected sample of descendants of people with dementia who have an increased risk of developing dementia [13]. To contribute to the development of a dementia risk reduction programme for descendants of people with dementia, the aim of the current study is to obtain insight into the knowledge, beliefs and attitudes towards dementia and dementia risk reduction among descendants of people with dementia. Fundamental elements can be captured to improve the willingness of middle-aged descendants of people with dementia to participate in a dementia risk reduction programme and adopt a healthier lifestyle. Moreover, by revealing areas for improvement, insight can be obtained on what factors a dementia risk reduction programme should focus on to enable health behaviour change.

## Method

### Participants

In this qualitative study, focus group discussions were used. The study population consisted of descendants of people with Alzheimer’s disease (AD), vascular dementia (VD) or mixed dementia diagnosed at hospital memory clinics in the northern part of the Netherlands. Twenty-four eligible participants of all adult ages and different educational levels were approached between February and June 2017 by medical specialists at the hospital memory clinic after diagnosing the individual’s parents with dementia. Subsequently, these individuals were invited to participate in a focus group discussion shortly after the diagnostic consultation (1–2 months after diagnosis) and received a flyer with more information about the study. In total, nineteen eligible participants were interested in participation of which four could not participate due to practical reasons. Eventually, fifteen participants participated in one of the focus group discussions. The aim was to include four to six participants in each focus group, which were also referred to as ‘mini groups’. This type of focus group gives the moderator the opportunity to gain more information from each individual and give more attention to the participants on this sensitive topic [11].

### Data collection and procedure

We applied a narrative interview approach with a topic guide specifically designed for this study that enabled discussion, clarification and verification of unanticipated themes [14]. A semi-structured topic guide based on the HBM [6] was used, aiming to identify the factors influencing health behaviour change for dementia risk reduction in adult children of patients with AD, VD or mixed dementia. The topic guide included questions on knowledge, beliefs and attitudes towards dementia risk assessment and dementia risk reduction (see Supplementary file 1). The moderator used open-ended questions to facilitate discussions and to provide the opportunity to the participants to talk freely.

All the focus group discussions were held in a private room at the Medical Faculty of the University of Groningen (Groningen, The Netherlands). The focus group discussions were facilitated by an experienced female moderator (EM, also an ethicist) assisted by a trained female researcher (JV) who observed and took notes during the focus group discussions. Each focus group session had a duration of sixty to ninety minutes and was audio recorded with the permission of the participants for later analyses. Before each focus group, all participants were asked to complete a short questionnaire, including questions on age, gender and educational level. Drinks and snacks were provided during the focus group discussions. Afterwards, all participants received a voucher of twenty euros. The Medical Ethics Commission of the University Medical Centre Groningen (UMCG) concluded that this study was not subject to the Medical Research Involving Human Subjects Act.

### Analysis

Qualitative content analysis was used to analyse the focus group data. Audio recordings from the focus group discussions were transcribed verbatim and analysed using Atlas-Ti version 8.1. Each transcript was analysed thoroughly, and when appropriate, a code was generated and assigned by two researchers independently (JV and RB). Moreover, a coding protocol was developed based on the analysis of the first transcript. Consensus was reached regarding the content of the codes by four researchers (JV, EM, RB and NS), which were used for the two remaining transcripts. Then, similar codes were grouped together and subsequently categorized into themes.

## Results

Three focus group discussions were conducted in April, May and June 2017 with four to six participants each to achieve data saturation. The participant characteristics are provided in Table 1. The age of the participants ranged from 26 to 61 years (mean 48.8, standard deviation (SD) 12.0), and 80% of the participants were female. The majority (80%) of the participants had a high educational level (see Supplementary file 2 for the definitions of the educational levels).

**Table 1:** Participant characteristics (*N* = 15)

Focus group	Respondent	Sex	Age range	Educational level
1	1	Female	20–30	High
1	2	Female	40–50	High
1	3	Female	40–50	Low
1	4	Male	50–60	Low
1	5	Female	30–40	High
1	6	Male	60–70	High
2	7	Female	50–60	High
2	8	Male	50–60	High
2	9	Female	60–70	Middle
2	10	Female	20–30	High
2	11	Female	40–50	High
3	12	Female	60–70	High
3	13	Female	50–60	High
3	14	Female	60–70	High
3	15	Female	50–60	High

Four themes were identified in the analysis, of which three themes emerged directly from the topic guide: 1) knowledge on dementia and dementia risk reduction, 2) beliefs and attitudes towards dementia risk assessment and dementia risk reduction and 3) the requirements for a dementia risk reduction programme. One theme was not foreseen in the topic guide but instead featured prominently in the analysis, namely, the experiences of having a parent with dementia, including the related practical and emotional consequences for oneself. Although the research aim was to evaluate the knowledge, beliefs and attitudes towards dementia and dementia risk reduction, participants first exchanged their experiences of having a parent with dementia before we were able to discuss the topics in our topic guide. Each theme is described in the following sections.

### Experiences of having a parent with dementia

Participants underlined the need to talk and share their experiences of having a parent with dementia with people who have similar experiences: *‘*For example, the participants perceived dementia as a severe disease and saw their parent becoming a different person: *‘I feel that it is a demeaning illness because, as a person, you are so different after getting sick’ (female, 50-60 years).* Nevertheless, the participants were relieved when their parent finally got diagnosed and finally they knew what their parent was suffering from. Having a diagnosis also improved the understanding of their parent. For instance, they can now accept that their parent is not able to do the things anymore the way they did before: *‘Yes, that is why I was ultimately relieved that it had been diagnosed, that I knew then, and then, I kind of resigned myself to it because certainly in the beginning, years ago, I thought, mum, please hurry up, what do you mean, you can’t find the way anymore?’ (female, 50–60 years).*

Having a parent with dementia has practical and emotional consequences. A practical consequence is taking care of their parent, which requires time: *‘Well, of course, I’m a busybody, I mean, as an informal carer. I visit on average two to three times a week, so yes, that is rather intense’ (female, 60–70 years).* An emotional consequence of having a parent with dementia is the anxiety to develop dementia. When a parent was diagnosed with dementia at a particular age, participants were afraid to be confronted with dementia at the same age: *‘My mother was diagnosed with Alzheimer’s when she was 57, and she died of it when she was 67 (…) me and my brothers, we sometimes talk about it; we are simply afraid that we may be confronted with it at the same age’ (female, 40–50 years).* Nevertheless, learning to cope with having a parent with dementia was more pressing than thinking about their own risk of developing dementia: *‘I am more concerned about my parents than about myself’ (male, 50–60 years).*

### Knowledge on dementia and dementia risk reduction

The general knowledge of dementia varied between participants, regardless of their age, gender and educational level. Several participants explained the use of dementia as an umbrella term: *‘Well, I think that dementia is an umbrella term, covering all those [types of dementia]’ (female, 60–70 years).* Some of the participants thought that Alzheimer’s disease is worse than “normal” dementia. A small number of participants even explained the pathology of dementia, although hesitantly: ‘*It’s to do with proteins in the brain, that the transmission of signals is poorer, and so on’ (female, 50–60 years).* Most participants were uncertain about the heritability of dementia*: ‘I’m not sure whether it is hereditary or not, or perhaps early-onset dementia is, I really don’t know’ (female, 60–70 years).* One participant was even hesitant to obtain information about the heritability of dementia, since she was afraid to find information she did not want to know.

Regarding their knowledge of dementia risk reduction, participants were initially uncertain whether the development of dementia later in life could be prevented or delayed. Therefore, non-modifiable risk factors were often mentioned first, such as age, genetics and family history. After encouraging them, participants also correctly guessed the majority of the currently known modifiable risk factors for dementia, such as poor diet and lack of cognitive activities. Participants also had suspicions and questions about other possible risk factors for dementia, such as sleeping behaviour, stress, traumatic experiences and mental wellbeing. Furthermore, several participants believed that a regular check of cholesterol, blood pressure and diabetes could also contribute to dementia risk reduction: *‘Yes, and what we can do about it? Well, be watchful and check often’ (male, 50–60 years).* All the identified risk factors by the group are presented in Table 2.

**Table 2:** Identified risk factors for dementia by the focus group participants

Non-modifiable risk factors	Modifiable risk factors	Modifiable risk factors (suspicions)
Age	Poor diet (e.g., salt)	Sleeping behaviour
Genetics	Physical inactivity	Stress
Family history	Smoking	Traumatic experiences
	Alcohol use	Mental wellbeing
	Cognitive activities	
	High cholesterol	
	Hypertension	
	Diabetes	
	Cardiovascular diseases	

The majority of the participants indicated that most of their knowledge was gained from the internet, family and friends or a caregiver in healthcare. Participants indicated that their general practitioner only provided minimal information about heritability: ‘*I have discussed it with my GP, who gave me very little information. He said we can do a test or something (…), but otherwise he didn’t give me much information’ (female, 40–50 years).* Overall, the participants were eager to receive more information on dementia and dementia risk reduction.

### Health beliefs and attitudes towards dementia risk assessment and dementia risk reduction

Initially, most participants believed that a dementia risk assessment is a genetic test that shows the chance of developing dementia later in life. Given that they were unaware or uncertain about the opportunity to reduce their risk of developing dementia, most participants were also uncertain whether they would want to have their dementia risk assessed. Some participants indicated that they would like to have their risk assessed and subsequently reduce their dementia risk, but they were uncertain about whether this was possible. Their beliefs and attitudes towards dementia risk assessment and dementia risk reduction are reflected in their motives to assess dementia risk and reduce their dementia risk, which are shown below.

The most frequently mentioned motive to assess dementia risk was the possibility of acting upon the outcome of a risk assessment: *‘I would only want it if you know you can do something about it because otherwise it’s just a dark cloud hanging over your head’ (female, 20–30 years).* Another motive was the optimism of having a treatment available in the future, so if necessary, this treatment could cure their dementia in the future. Several other motives to assess and reduce dementia risk were mentioned after providing the participants with information regarding dementia risk reduction. One of these motives was to adopt healthy behaviour for dementia risk reduction to age healthily. Some said they would do anything to turn the tide of the development of dementia and grow old in good health. Another participant added that there is no harm in trying and considered to take the information more seriously*: ‘Yes, I feel like this can’t really hurt. Maybe there is something in what they say. I don’t know , do something with your life, drink less alcohol. I don’t know, but well, it doesn’t hurt to try’ (female, 20–30 years).* Some participants found it already valuable to obtain insight into their health and lifestyle and just wanted to know everything about their health, even when it was not positive. Another motive was ‘to have self-control’, for instance by anticipating the results of a dementia risk assessment: *‘I very much want to stay in charge (…) that is most important to me. That’s why I would like to know (…). I am the kind of person who would opt for euthanasia at the final* stage’ (*female, 60–70 years*). Finally, one participant also mentioned their current cognitive health as a motive to adopt a healthy behaviour for dementia risk reduction: *‘I’d say yes because I forget a lot of things even now; I sometimes wonder what I did this morning’ (female, 60–70 years).*

Nevertheless, participants also mentioned several motives not to assess and reduce their dementia risk. A frequently mentioned motive not to assess dementia risk was that they are still young, and this would be something to consider in the future. However, one of the participants noticed that it could be possible to suffer from dementia already at her current age: *‘But, yes, that’s a bit funny. I think I’m [only] 60, but that’s nonsense, of course because there were 60-year-olds in my mother’s nursing home’ (female, 60–70 years).* Furthermore, the participants indicated that the outcome of a dementia risk assessment would cause restless feelings or anxiety given that it is unknown when symptoms will appear and how severe the symptoms will be. They also indicated that the outcome of the dementia risk assessment does not provide certainty that they will or will not develop dementia and that a healthy lifestyle is no guarantee to prevent dementia: *‘no matter how busy you are, those very active people, they get it too’ (female, 60–70 years).* Finally, the participants believed that the ultimate choice is a balance of interest between enjoying moments in life and having a healthy lifestyle: *‘You have to weigh up the interests, I think. So I think that I would consider something like, I am enjoying myself so much now, I will have a drink now and then maybe have a week less [to live] later on’ (female, 50–60 years).*

### Requirements for a dementia risk reduction programme

The participants expressed their need for more information on dementia and dementia risk reduction and would like to receive this information in a dementia risk reduction programme. However, their choice to participate in a dementia risk programme also depends on the content of the programme, intensity of the programme, type of advice given in the programme, outcome measure of the programme and specific functions of the programme. They mentioned several requirements for a dementia risk reduction programme. First, the programme should be a central point of reliable, clear and up to date information about dementia and dementia risk reduction. Second, the programme should offer regular health check-ups, which should reveal room for improvement in relevant lifestyle factors they can act upon: *‘if you really get the result like, “yes you will get it”, that is different from “maybe you can do something about this”’ (female, 20–30 years).* Subsequently, participants would like to receive personalized lifestyle advice and not general information that is applicable to everyone: *‘I think if it’s about general things like I just heard about (…), then I think that’s not something new for me, you know, (…), so I think then it must really be a specific thing for me, like this is your individual chance and you really have to do this very differently’ (female, 30–40 years).* Nevertheless, participation in the programme should not be too time consuming, since they also have a job, a parent to take care of and other activities in their lives. Further, participants would like to have the possibility to share information with their siblings. Finally, in order to increase motivation to stay in the programme, participants mentioned that it should enable participation without the help of healthcare providers, should be easily accessible, should provide regular reminders and it should not cause guilt feelings when not adhering to the advice. See Table 3 for an overview of the requirements.

**Table 3:** Requirements for a dementia risk reduction programme

Requirements	
Central point of reliable, clear and up to date information about dementia and dementia risk reduction	
Regular check-ups with an easy interpretable outcome measure and amenable for acting on	
Personalized lifestyle advice, including the benefits of adhering to the advice	
Not too time consuming. The intensity of the programme should not avert enjoying life next to a job and care of parent(s)	
Possibility to share information with siblings	
Easily accessible (e.g., without having to ask the general practitioner)	
Regular reminders by for example text messages	
Adopting a healthy behaviour is their own responsibility and independently performed without the help of healthcare providers	
Should cause no guilt feelings when not adhering to the lifestyle advice	

## Discussion

Although the research aim was to evaluate the knowledge, beliefs and attitudes towards dementia and dementia risk reduction among descendants of people with dementia, our findings demonstrate that individuals with a parent with dementia feel the need to share their experiences on how to cope with a parent with dementia with their peers and that their worry about their own risk of developing dementia was inferior to this need. Furthermore, initially, the participants were unaware or uncertain about the possibility of reducing the risk of developing dementia, resulting in uncertainty regarding whether they would like to assess their dementia risk. Although the participants identified a large number of modifiable risk factors as a group, they were eager to receive more information on dementia and dementia risk reduction. By sharing their experiences of having a parent with dementia and their knowledge of dementia, the participants adopted a more positive attitude towards participation in a dementia risk reduction programme and provided important elements for future dementia risk reduction programmes.

### Sharing experiences of having a parent with dementia

In the current study, the participants underlined the importance of sharing their experiences of having a parent with dementia with individuals who have had similar experiences. Sharing experiences of having a parent with dementia seemed to be a prerequisite to thinking about their own health and dementia risk and facilitated movement between the pre-contemplation phase and the contemplation phase of behaviour change [15]. Therefore, it is important to incorporate interactions between peers, for example, group-based interventions. This setting might encourage individuals to participate and adhere to the programme. To the best of our knowledge, the FINGER (Finnish Geriatric Intervention Study to Prevent Cognitive Impairment and Disability) trial is the only dementia risk reduction trial to date that provided group-based as well as individual interventions that significantly reduced dementia risk by improving or maintaining cognitive functioning [16]. In light of our results, the group-based interventions of this trial potentially primarily contributed to the effectiveness of the multi-domain intervention.

### Knowledge on dementia and dementia risk reduction

We found that knowledge on dementia and dementia risk reduction was limited, even among descendants of people with dementia. Most participants believed that a dementia risk assessment is a genetic test that shows the chance of developing dementia later in life. At first, the participants were uncertain whether it was possible to modify their risk for developing dementia later in life. Nevertheless, the participants in the current study eventually identified several modifiable risk factors for dementia as a group, which included the majority of the currently known modifiable risk factors for dementia, such as cardiovascular diseases [1, 2]. The risk factors loneliness, obesity and renal dysfunction were not mentioned by the group. In the Netherlands, approximately 11% of the general population identified renal dysfunction as a risk factor for dementia, indicating that the majority are unaware of renal dysfunction being a risk factor for dementia [17]. The participants in the current study also had suspicions about whether sleeping behaviour, stress, traumatic experiences and mental wellbeing were modifiable risk factors for dementia. Although strong and sufficient evidence for these factors is still lacking, some studies support that these factors might play a role in the development of dementia [18–20].

Furthermore, at first, the participants were hesitant about assessing dementia risk without a treatment in sight due to their unawareness of the possibility of reducing dementia risk. This lack of knowledge forms a barrier towards lifestyle changes for dementia risk reduction. Individuals with more knowledge about dementia and dementia risk reduction might be more likely to adopt healthy behaviour. Therefore, promoting dementia awareness should especially be considered for descendants of people with dementia since this group at risk for dementia might be more receptive to health behaviour change. Improved knowledge about dementia and dementia risk reduction is not only helpful for reducing dementia risk, but could also be helpful for dealing with dementia related needs of the parent. Therefore, it is helpful for both the descendant and the parent with dementia.

### Health beliefs and attitudes towards dementia risk assessment and dementia risk reduction

The participants in the current study perceived dementia as a severe disease and worried about developing dementia themselves. Previous literature has shown that individuals with a parental family history have a higher perceived risk of developing dementia than individuals without a parental family history [21–23]. According to the HBM, perceived risk is one of the determinants influencing the probability of adopting healthy behaviour [6]. Therefore, our hypothesis was that descendants of people with dementia are more receptive to adopting healthy behaviour for dementia risk reduction. However, despite their increased motivation to adopt healthy behaviour, our findings show that having a parent with dementia causes anxiety and might form a barrier to assess their risk and adopt healthy behaviour. Previous findings about whether having a family history has a positive effect on the motivation to adopt a healthy lifestyle appear to be contradictory [21, 24–26]. Two studies did not identify a difference in risk-reducing behaviour (e.g., trying to stop smoking, increasing physical activity) between individuals with and without a family history of cardiovascular disease [24, 26]. However, two other studies demonstrated that a family history of diabetes, anxiety, depression and high blood pressure is positively associated with risk awareness and risk-reducing behaviour [21, 25]. The self-perceived risk (e.g., perceived severity and perceived susceptibility) of developing a certain disease might mediate the association between having a family history and interest in health education to adopt a healthy behaviour [21]. Further, participants were afraid that the outcome of a dementia risk assessment might cause restless feelings or anxiety, since it does not provide certainty that they will or will not develop dementia later in life and a healthy lifestyle is not a guarantee that they will not develop dementia. This may suggest that focussing on maintaining optimal cognitive health instead of reducing dementia risk is preferred.

### Strengths and limitations

To our knowledge, this was the first study that explored the knowledge, beliefs and attitudes towards dementia and dementia risk reduction among a selected sample of descendants of people with dementia. A major strength of this study is that it explored not only the knowledge but also the beliefs and attitudes of these individuals towards dementia and dementia risk reduction. Adequate knowledge is not sufficient for health behaviour change. Also positive health beliefs and attitudes towards dementia and dementia risk reduction are needed. With this study, we provided insight in what health beliefs and attitudes towards dementia and dementia risk reduction need to be improved in order for dementia risk reduction programmes to be effective. Another major strength is that we used focus group discussions, which are recommended to explore beliefs about health and disease [27]. Due to this study design, we were able to identify a finding that we did not anticipate in the topic guide. In addition, participants could share their opinion and react on each other’s comments, leading to a discussion. This provided us with insightful information that we might not have collected using individual interviews. However, this study had certain limitations. The recruitment of participants was difficult. Not surprisingly, mainly highly educated individuals and females were included. Moreover, the recruitment setting may have led to selection bias for several reasons. First, females are more often informal caregivers and therefore accompany their parent more often to the hospital memory clinic [28]. Second, mainly patients with complex types of dementia visit the hospital memory clinic since patients need to be referred by their general practitioner [29]. Therefore, the study sample might not be representative of all descendants of people with dementia in the Netherlands. Most of the participants are highly educated. Knowledge about dementia and dementia risk reduction might be worse in lower educated individuals, resulting in different beliefs and attitudes towards dementia and dementia risk reduction.

### Implications

These findings can be used in the development of dementia risk reduction programmes for descendants of people with dementia. Our findings strongly point to the importance of incorporating the possibility of exchanging experiences related to having a parent with dementia with individuals who have had similar experiences in a dementia risk reduction programme. It seemed that sharing experiences of having a parent with dementia is a prerequisite for offspring to think about their own health and dementia risk. Additionally, descendants of people with dementia made several recommendations about which other elements should be included in a dementia risk reduction programme (see Table 3). Based on these recommendations, the online lifestyle programme for the Demin study was developed [30]. This programme consisted of: 1) a dementia risk assessment on five measurement moments during 1 year follow-up (online questionnaires, physical examination and blood sample) and 2) an online tailor-made lifestyle advice regarding protective (Mediterranean diet, low/moderate alcohol consumption, high cognitive activity) and risk factors (physical inactivity, smoking, loneliness, cardiovascular disease, hypertension, high cholesterol, diabetes, obesity, renal dysfunction, depression) for dementia. The outcome of the dementia risk assessment was indicated by the Lifestyle for Brain Health (LIBRA) score in which each protective and risk factors for dementia was categorized into one of the following categories: 1) keep this up, 2) room for improvement or 3) remember to manage well [31]. This type of outcome measure is easy interpretable and amenable for acting on. To improve the knowledge of the potential participants, we provided general information about dementia and dementia risk reduction on the Demin website (www.demin.nl), in plain text and spoken animations. Furthermore, the participants received tailor-made lifestyle advice, including information about the protective and risk factors for dementia, its association with dementia and recommendations how to improve their lifestyle with regard to that specific protective or risk factor. Unfortunately, it was not possible within the Demin study to incorporate the possibility for social contact between participants due to its construct (online lifestyle advice). However, participants had the opportunity to invite their siblings to participate in the study too. In the Demin study the uptake and effectiveness of this online lifestyle programme was investigated among individuals with a parental family history of dementia [30]. When the opportunity to share experiences of having a parent with dementia is also included in future dementia risk reduction programmes, the willingness to participate in a dementia risk reduction programme and the effectiveness in adopting and maintaining healthy behaviour among descendants of people with dementia might be further improved.

Our findings also support reinforcing knowledge about dementia and increasing the awareness of the opportunity to reduce dementia risk through a healthy lifestyle. More knowledge and awareness can contribute to more positive health beliefs and attitudes towards dementia risk reduction. For example, this increase in knowledge could be achieved through a targeted national mass media campaign aiming to motivate individuals to address their personal risk factors. As general practitioners are often the first point of contact for people who are concerned about their health and dementia risk, they should be educated about the opportunity to reduce the risk of developing dementia and methods to use this information to inform descendants of people with dementia properly.

### Recommendations for future research

First, evaluating the knowledge, beliefs and attitudes of less educated descendants of people with dementia would be a valuable addition for future research since there is more room for improvement regarding lifestyle changes for dementia risk reduction. Subsequently, we encourage the development of dementia risk reduction trials for descendants of people with dementia, including the possibility of exchanging experiences with individuals who have had similar experiences to improve recruitment and to be effective in adopting healthy behaviour for dementia risk reduction.

## Conclusion

Sharing experiences of having a parent with dementia seemed a prerequisite for thinking about one’s own risk of developing dementia and participating in a dementia risk reduction programme. Knowledge of dementia and dementia risk reduction is limited. Due to the unawareness of the possibility of reducing dementia risk, the participants were hesitant about assessing their own dementia risk without a treatment in sight. Sharing information about risk factors for dementia and the importance of a healthy lifestyle could change people’s perception of dementia risk assessment and their willingness to participate in a health behaviour programme for dementia risk reduction. Therefore, education on dementia and dementia risk reduction is needed.

## Supplementary Information

**Additional file 1: Supplementary Table 1.** Focus group topics and illustrative questions. **Supplementary file 2.** Definitions of low, middle, and high level of education based on the International Standard Classification of Education.

## Data Availability

The data collected during this study will be available from the corresponding author upon reasonable request.

## Abbreviations

DRR: Dementia Risk Reduction
HBM: Health Behaviour Model
AD: Alzheimer’s Disease
VD: Vascular Dementia
UMCG: University Medical Centre Groningen
SD: Standard deviation
FINGER: Finnish Geriatric Intervention Study to Prevent Cognitive Impairment and Disability
