# Supplementary material for: Knowledge, health beliefs and attitudes towards dementia and dementia risk reduction among descendants of people with dementia: a qualitative study using focus group discussions
Source: BMC Public Health. 2021 Jul 7;21:1344. doi: 10.1186/s12889-021-11415-2 (PMC8265097; doi:10.1186/s12889-021-11415-2)
Supplement: Supplementary file 1 — Additional file 1: Supplementary Table 1. Focus group topics and illustrative questions. Supplementary file 2. Definitions of low, middle, and high level of education based on the International Standard Classification of Education. [file 12889_2021_11415_MOESM1_ESM.docx]

**Supplementary table 1** Focus group topics and illustrative questions

| **Topic** | **Example questions** |
| --- | --- |
| Personal involvement | Why did you decide to participate in this focus group? |
| General knowledge about dementia | What is dementia? Thinking of ‘dementia’, what comes to your mind? |
| Knowledge about dementia risk reduction  Source of information  Perceives susceptibility  Motives for dementia risk assessment  Motives for dementia risk reduction  Requirements | Do you think the risk to develop dementia can be reduced? and what do you think are risk factors for dementia?  Where did you get this information from?  What is your likelihood of developing dementia?  What would motivate or stop you to assess your risk of developing dementia?  What would motivate or stop you to change your health behaviour and lifestyle to reduce your risk of dementia?  What should an online dementia risk reduction program look like? |
| Other | Do you have any other thoughts or views you would like to share? |

**Supplementary file 2:** Definitions of low, middle, and high level of education based on the

| **Highest level of completed education** | **Definition** |
| --- | --- |
| Low | Less than primary education Primary education Lower secondary education |
| Middle | Upper secondary education Post-secondary non-tertiary education |
| High | Short-cycle tertiary education Bachelor or equivalent education Master or equivalent education Doctoral or equivalent education |

International Standard Classification of Education.
